# A study on the spatial distribution characteristics and driving factors of traditional villages in the southeast coast of China: A case study of Puxian, Fujian

**DOI:** 10.1371/journal.pone.0303746

**Published:** 2024-06-07

**Authors:** Xiaoxue Lu, Zhuo Peng, Yuchen Zhou, Yanqiu Xie, Zujian Chen

**Affiliations:** 1 College of Landscape Architecture and Art, Fujian Agricultural and Forestry University, Fuzhou, China; 2 Chengdu University of Technology, Chengdu, China; 3 Jiangxi Agricultural University, Nanchang, China; National Taiwan Normal University - Gongguan Campus, TAIWAN

## Abstract

Traditional villages are the common historical and cultural heritage of all mankind. With the intensification of urbanization, the continuation of traditional villages and the inheritance of historical and cultural heritage are facing risks. The research on the driving factors affecting the spatial distribution characteristics, heterogeneity and human land interaction of traditional villages provides a new idea for the protection of traditional villages. This study takes 137 traditional villages in Puxian area, a typical cultural area in the southeast coast, as the research object, analyzes the spatial distribution pattern of traditional villages by using spatial analysis method, and selects 13 factors to analyze the main driving forces and interaction mechanisms through geographical detectors. The results show that: (1) Puxian traditional villages are clustered and distributed, and the distribution among counties is uneven, mainly in the state of "one cluster and many scattered points" with more coastal areas and less mountainous areas. (2) Puxian traditional villages are mainly affected by many factors such as nature, space, society and culture. They are more densely distributed in areas with rich cultural heritage, fertile land, flat terrain, suitable climate, close to water systems, developed transportation, backward economy and dense population. (3) Cultural factors are the primary factors affecting the spatial distribution of traditional villages, the order of driving factors’ explanatory power is: intangible cultural heritage (0.5160) > protected cultural relic units (0.3591) > distance from railway (0.3255) > night light remote sensing (0.3179) > elevation (0.3012) > population density (0.2671) > slope (0.2032) > soil type (0.1804) > precipitation (0.1750) > temperature (0.1744) > land use (0.1492) > distance from river (0.0691)>distance from highway (0.0530). The interaction of intangible cultural heritage, protected cultural relic units and distance from the railway is the dominant factor for the spatial differentiation of traditional villages. Among them, the interaction of intangible cultural heritage∩distance from the railway is the strongest, and the q-value is 0.79, which proves that the interpretation ability of the two factor model is much higher than that of the single factor model. The results of this study reflect that traditional villages and nature, space, society and culture are interdependent, so the protection of traditional villages should be adapted to local conditions.

## 1 Introduction

As a country with a long civilization history of thousands of years, China has bred many traditional villages on a vast land. Traditional villages are villages that were formed earlier, contain rich cultural and natural resources, have specific historical, cultural, social, scientific, economic and artistic values, and need to be protected [1]. They have profound cultural deposits and distinctive regional characteristics. China has a long history, vast land and a large number of traditional villages, which are the most distinctive part of villages [2, 3]. However, with China’s rapid urbanization (64.72%) [4] and industrialization [5], traditional villages are facing serious problems such as loss of local characteristics, weak local identity among residents [6], population outflow, and ecological damage [7, 8]. From 2002 to 2022, the number of natural villages decreased from 3.5 million to 2.4 million, and the number of administrative villages decreased from 700000 to less than 550000, including a large number of traditional villages [2], which hinders the protection and development of villages. In response to this phenomenon, China has currently comprehensively promoted the key work of rural revitalization, which has made rural development in China increasingly valued.

With the development of geographic information system (GIS) and the wide application of multivariate statistical analysis, it is possible to carry out research from the national [9–11], provincial [12, 13], special geographical areas [14–16] and other macro scales. Jiao Jinying et al. (2022) quantified the factors affecting the spatial distribution of 2424 traditional villages in the Henan section of the Yellow River Basin, and found that natural factors such as rivers and topography were the main factors, followed by human factors [17]; Li J et al. (2022) studied the spatial characteristics of 98 traditional villages along the Han silk road in Ningxia and found that the central settlements and transportation were the main influencing factors, and the influence of human factors was significantly greater than that of natural factors [18]. To sum up, the influencing factors and formation mechanism of the spatial layout characteristics of traditional villages are complex, mainly affected by natural environmental factors, socio-economic factors and historical and cultural factors. At the same time, the spatial differences of different regions will also lead to different research results. However, at present, the research on the spatial distribution of traditional villages and its driving factors is mostly from the macro perspective, less from the perspective of city and cultural area. At the same time, the protection of local villages at the micro scale is more targeted.

In addition, most of the current research methods are based on ArcGIS spatial autocorrelation analysis, combined with residual analysis or correlation analysis; However, residual analysis cannot quantify the analysis factors [19], while correlation analysis can quantify but not express spatial heterogeneity. It is suggested that [20]. By comparison, the geographical detector studied by Wang Jinfeng et al. [21] evaded the impact of multivariable collinearity, solved the impact of a single factor on the dependent variable and the impact of the interaction of two factors, and quantitatively determined the impact of each factor on the spatial heterogeneity of traditional villages without considering linearity. Because it can measure the contribution of each factor more intuitively, quickly and effectively [22], it overcomes the limitations of traditional methods in the analysis of categorical variable [23], and does not need strong model assumptions. It has been widely used in various fields such as regional development [24], ecological environment [25], and land use [26, 27]. It has gradually been applied in the field of traditional village spatial distribution, For example, Guan Zhongmei (2017) and others used nuclear density analysis, geodetectors and other methods to discuss the spatial-temporal pattern and causes of the distribution of traditional villages in the Central Plains Economic Zone [28]; Xue Mingyue (2020) and others analyzed the spatial differentiation characteristics and influencing factors of traditional villages in the Yellow River Basin by using geographical analysis tools and geodetectors [29]; Yang Yan (2021) and others analyzed the spatial differentiation and influencing factors of ethnic traditional villages in Guizhou Province with the help of geodetectors and other methods [30]. Existing studies have shown that there are many types of spatial distribution of traditional villages, such as agglomeration type, random type and uniform discrete type, with significant regional differences. The differentiation is affected by the complexity, diversity, nonlinearity and heterogeneity of each influencing factor [31]. Basic qualitative analysis and single factor exploration are difficult to accurately quantify the interaction between the factors affecting the distribution of traditional villages [19]. Therefore, exploring the spatial distribution characteristics of traditional villages through reasonable statistical analysis means can master the human land interaction mechanism of villages and establish scientific protection and continuation policies, which is of great significance to the construction of ecological civilization and the protection of local cultural heritage and cultural diversity [32].

Puxian area is located in the southeast coast of China and has unique advantages in marine resources. It has been an important node of the maritime Silk Road and a transit station for Sino foreign maritime trade since ancient times. Puxian area has a complex and diverse terrain, with mountains, plains, coasts, islands and other terrain from west to East. At the same time, it has distinctive local cultural characteristics, has a number of world-class intangible cultural heritage, and now has 579 cultural relics protection units at all levels. It is also one of the main components of traditional villages in Fujian. According to the comprehensive situation of dialect distribution, regional culture and natural geographical conditions, Professor Dai Zhijian [33] divided the ancient architecture in Fujian Province into six regions, and Puxian dwellings were classified into a single category, which shows that Puxian is typical in terms of region and culture. Therefore, in order to grasp the spatial distribution characteristics and driving factors of traditional villages in meso or micro scale cultural areas, this paper takes Puxian area as the research area, and uses geographic information technology and geodetectors to analyze the impact of natural, spatial, social, cultural and other factors on the spatial distribution characteristics, driving factors and interactions of traditional villages in micro scale cultural areas. The results of this study can provide references for mastering the man land interaction mechanism of traditional villages and establishing scientific protection and continuation policies.

## 2 Materials and research methods

### 2.1 Overview of the study area

**Fig 1:**
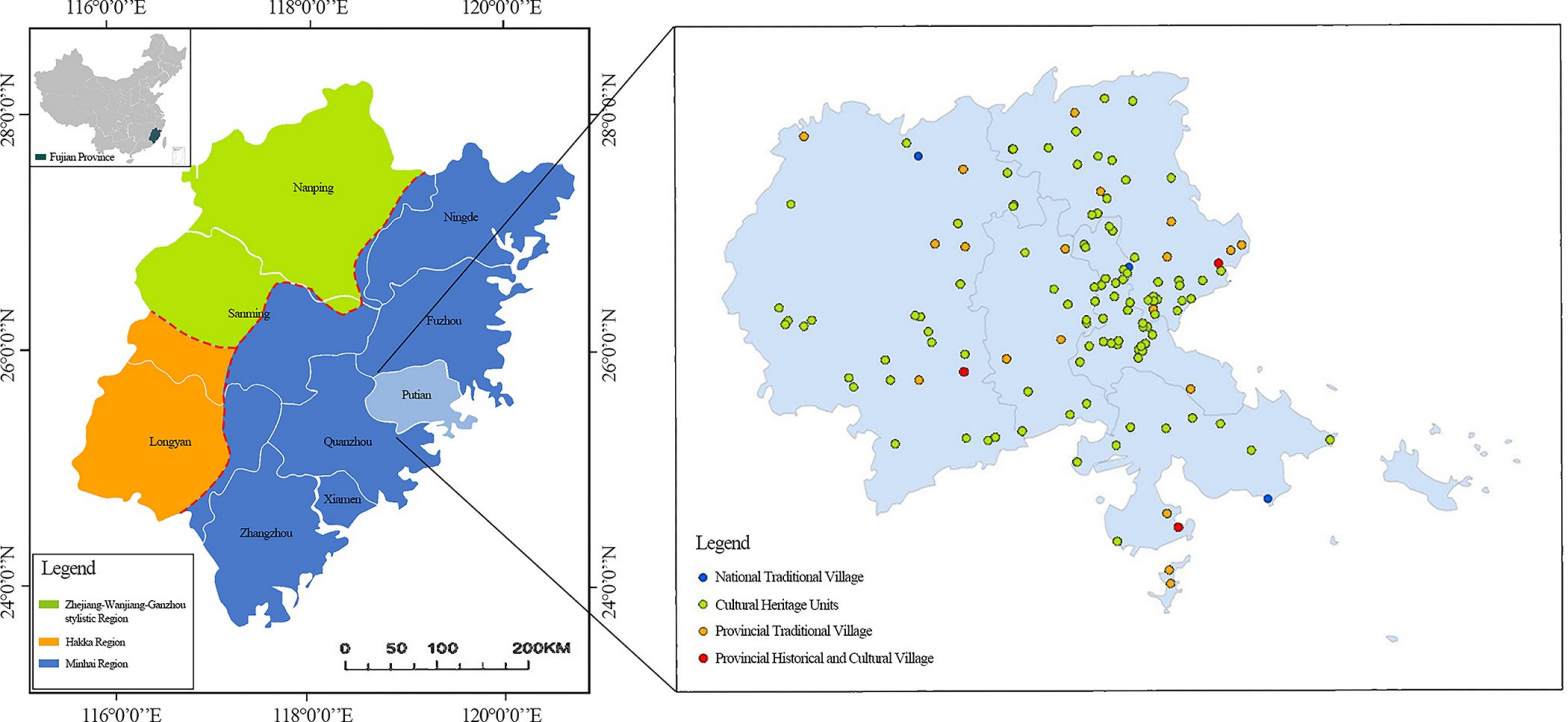
Spatial distribution of Puxian traditional villages.

Puxian (latitude 24°59′-25°46′N, longitude 118°27 ′-119°56′E) refers to Putian City and Xianyou County (Fig 1). It now governs four districts and one county (Licheng District, Chengxiang District, Hanjiang District, Xiuyu district and Xianyou County). Puxian is located in the second uplift zone of the Neocathaysian giant structure in East Asia, the eastern subsidence zone and the eastern end of the Nanling giant latitudinal structural system. The terrain in Puxian is high in the northwest and low in the southeast. The cross section is saddle shaped, backed by the Daiyun mountains and facing the Taiwan Strait. The land area is 4200 hm^2^ and the sea area is 11000km^2^, The annual average temperature is between 16°C and 21°C, and the annual average precipitation is between 1000 mm and 2300 mm. Puxian has been the political, economic and cultural center of central Fujian since ancient times. It is the birthplace of Chinese Mazu culture and an important trade port for foreign merchants on the maritime Silk Road [34].

### 2.2 Data sources

#### 2.2.1 Dependent variable

This study obtained a total of 37 national and provincial-level traditional villages and historical and cultural villages from the official website of the Ministry of housing and urban rural development of China. In addition, there are still many villages that have not been rated but have been registered by cultural relics protection units or township management departments, including cultural relics and historic sites and cultural relics protection units [35], and according to data access and field research, a total of 100 villages with traditional village culture were added (Table 1). The average nuclear density (expressed by the dependent variable y) can clearly reflect the distribution pattern of traditional villages in the study area. Baidu map API coordinate picker was used to select the longitude, latitude and longitude values of 137 traditional villages, and the administrative boundary vector data of the study area was obtained from the 1.4 million vector map information database provided by the national basic geographic information center (http://www.ngcc.cn/ngcc/) (Fig 1).

**Table 1:** Supplementary standards for traditional villages.

Number	Indicator type	Contents	Defining criteria
1	Geographical scope	Distribution scope of villages	Puxian four districts and one county
2	Village establishment time	Village site selection, formation time, and the earliest existing buildings	Before the Republic of China
3	Historical and cultural values	Site selection and construction	The site selection and construction of villages have typical Puxian cultural characteristics and historical background, and can reflect the historical, cultural, scientific, social and artistic values contained in the site selection of Puxian villages
4	Traditional pattern	Village and environment	The landscape pattern, natural environment, historical features and economic and social system of the village are improved
5	Historical relics	Architectural remains and historical environmental elements	The number of traditional buildings accounts for 60% of all buildings in the village
6	Traditional architecture style	Preservation of buildings and building complexes	The existing traditional buildings (clusters), building details and the original appearance of the surrounding environment of the buildings are well preserved, and there are native residents living there
7	Traditional lifestyle	Production and lifestyle	Based on the traditional farming production mode, it continues the local traditional skills, wedding and funeral customs and festivals, and retains the clan concept

### 2.2.2 Independent variable

Previous studies have considered the impact of natural, demographic, economic and traffic factors. In order to establish a more complete index system, this study incorporated the regional cultural factors into the analysis model. The factors selected in this study mainly include four categories.

#### (1) Natural factors

The terrain data [36] was downloaded from the official website of the Space Shuttle Radar topographic mapping mission (SRTM) (http://www.gscloud.cn/). The digital map elevation with DEM data resolution of 30 m is obtained by splicing, projection and clipping, and obtaining elevation and slope information using 3D analysis tools in ArcGIS10.5 (recorded as variables X6 and X9). Soil types [37] were obtained from the resource and environmental science and data center of the Chinese Academy of Sciences (http://www.resdc.cn/) (recorded as variable X4). Temperature and precipitation data [38] were obtained from the national Qinghai Tibet Plateau scientific data center (http://data.tpdc.ac.cn/) (recorded as variables X7 and X10).

#### (2) Spatial factors

The distance between traditional villages and rivers and roads can reflect their spatial accessibility and convenience. Water source is the key to the layout of traditional villages, and it is also the main water source for production and living. The density of road network is closely related to the terrain. Road and railway data [39] were obtained from the 1:100000 Public Edition Basic Geographic Information Data (http://www.webmap.cn/). Water system data [40] were obtained from the resource and environmental science and data center of the Chinese Academy of Sciences (http://www.resdc.cn/). Using Euclidean distance (recorded as variables X1, X2, and X3) in ArcGIS10.5, calculate the distance between the village and these two features.

#### (3) Social factors

Nighttime lighting is a representation of human activities, that is, the brighter the nighttime lighting, the higher the GDP level. The economy promotes the development of villages. The nighttime lighting can clearly reflect the economic development level of Puxian area. The nighttime lighting data [41] was from the resource and environmental science and data center of the Chinese Academy of Sciences (http://www.resdc.cn/) (recorded as variable X11). The total population is an important factor affecting the spatial distribution of traditional villages created by people-oriented. The residents have created settlements, and the historical features will change with the location of the village. The population data [42] were from the world pop and the statistical yearbook (http://www.worldpop.cn/, http://tjj.fujian.gov.cn/) (recorded as variable X8). Land use is a kind of intervention activity conducted by human beings for a certain purpose according to the natural and social attributes of national land resources. It is the embodiment of human social activities in villages. Land use data [43] was obtained from the resource and environmental science and data center of the Chinese Academy of Sciences (http://www.resdc.cn/) (recorded as variable X5).

#### (4) Cultural factor

Intangible cultural heritage is the embodiment of intangible traditional culture, and cultural relics protection units are tangible carriers of intangible culture to a certain extent. The data of intangible cultural heritage [44] came from China’s intangible cultural heritage website (https://www.ihchina.cn/). The data of cultural relics protection units were from the list of the first and eighth batch of cultural relics protection units published by the State Council (recorded as variables X12 and X13).

### 2.3 Research method

#### 2.3.1 Spatial distribution characteristics

*(1) Nearest neighbor index method*. The nearest neighbor index method [45] is to study the proximity of traditional villages to each other in space in the form of point targets. If R = 1, the distribution of Puxian traditional villages is random; If R>1, the distribution of villages is uniform; If R<1, the villages show aggregation distribution characteristics.

$$R=\frac{\bar{r_{1}}}{r_{E}}=2\sqrt{D}\times \bar{r_{1}}\bar{\gamma_{E}}$$ (1)

$$=\frac{1}{2\sqrt{n/A}}=\frac{1}{2\sqrt{D}}$$

Where, $\bar{r_{1}}$ Represents the actual nearest distance, $\bar{r_{E}}$ Represents the theoretical nearest distance, n represents the number, A represents the area of the study area, D represents the point density value, and the ratio of the actual nearest distance to the theoretical nearest distance is R.

*(2) Geographic concentration index*. Geographic concentration index [46] is an important parameter for further analysis of the spatial distribution and concentration degree of the research object. The value of G ranges from 0 to 100. The larger the value of G, the more concentrated the distribution of traditional villages.

$$G=100\sqrt{\sum_{i=1}^{n}{\left(\frac{x_{i}}{T}\right)}^{2}}$$ (2)

Where, the geographical concentration index of traditional villages represents G, X_i_ Represents the number of traditional villages in the ith county (district) in Puxian area, T represents the total number of traditional villages, and n represents the total number of counties (districts).

*(3) Spatial Gini coefficient*. The spatial Gini coefficient [47] is an important indicator of the discrete distribution in a study area in geography. Gini coefficient is between 0 and 1. The greater the coefficient is, the higher the degree of concentration is. Be able to compare the differences in the spatial distribution of geographical elements.


$$Gini=-\sum_{i=1}^{N}\frac{P_{i}\;\ln\;P_{i}}{\ln\;n}$$ (3)

C=1−Gini

*(4) Imbalance index*. The imbalance index [48] reflects the degree of distribution balance of Puxian traditional villages. S is between 0 and 1. If S = 0, it means that traditional villages are evenly distributed in each county; If S = 1, it means that traditional villages are clustered and distributed in a county.

$$S=\frac{\sum_{i=1}^{n}Y_{i}-50(n+1)}{100n-50(n+1)}$$ (4)

Where, n represents the number of Puxian County, Y_i_ Represents the cumulative percentage of the i-th place after ranking the proportion of traditional villages in counties and districts in the region in descending order.

*(5) Nuclear density estimation method*. The nuclear density estimation method [10, 49] is a general measurement index of spatial regional aggregation degree. Use mobile units to estimate the density of point or line patterns. Taking the position of each sampling point as the center, the kernel density function is used to calculate the density contribution value of each grid cell within the specified range of each sampling point, and the density at the edge of the range is 0.

$$f(x)=\frac{1}{nh}\sum_{i=1}^{n}k(\frac{x-x_{i}}{h})$$ (5)

Where is the kernel density estimation formula; $k(\frac{x-x_{i}}{h})$ is a kernel function; H is bandwidth (H > 0); N is the point within the threshold; x−x_i_ is the distance between the estimated point and the event point.

#### 2.3.2 Spatial heterogeneity

Geodetector [29, 50] is a method to detect the spatial differences of geographical elements and reveal the causes of spatial differences, such as factor detection, interactive detection, risk detection, ecological detection, etc.

*(1) Factor detector*. The effect intensity of each factor on spatial difference was analyzed

$$q=1-\frac{\sum_{h=1}^{L}N_{h\sigma_{h}^{2}}}{N\sigma^{2}}$$ (6)

In the formula, q-value is the index to measure the detection power of the independent variable, and the value is between 0 and 1. The closer to 1, the greater the influence of the factor; L is the stratification of independent variable or dependent variable; *N*_*h*_ and $\sigma_{h}^{2}$ Are the number of units and variance of layer h, respectively; N and σ^2^ They are the number of units and variance of the whole.

*(2) Interaction detector*. By determining the q-value of the interaction between two different independent variables, the influence of the interaction between independent variables on the dependent variable can be determined $q(X_{a}\cap X_{b})<min[q(X_{a}),(X_{b})]$ is the nonlinear weakening of the interaction; $min[q(X_{a}),(X_{b})]<q(X_{a}\cap X_{b})<max[q(X_{a}),(X_{b})]$ is a single factor nonlinear weakening; $q(X_{a}\cap X_{b})>max[q(X_{a}),(X_{b})]$ is a double factor enhancement; $q(X_{a}\cap X_{b})>q(X_{a})+q(X_{b})$ is an independent interaction; $q(X_{a}\cap X_{b})>q(X_{a})+q(X_{b})$ is non-linear enhancement.

*(3) Risk area detection*. It is used to test whether there are significant differences between the spatial patterns represented by the average value and the sub regions divided by category or hierarchical variables. t statistics is used to test:

$$t_{{\bar{y}}_{h=1}-{\bar{y}}_{h=2}}=\frac{{\bar{Y}}_{h=1}-{\bar{Y}}_{h=2}}{{\left[\frac{Var({\bar{Y}}_{h=1})}{n_{h=1}}+\frac{Var({\bar{Y}}_{h=2})}{n_{h=2}}\right]}^{1/2}}$$ (7)

In the formula, ${\bar{Y}}_{h}$ is the mean value of the attributes in the sub region h; *Var* is variance; *n*_*h*_ is the number of samples in sub region h; The statistic t approximately obeys the student’s t distribution, and the greater the t value, the greater the influence of this factor on the spatial differentiation of traditional villages.

*(4) Ecological detector*. It is used to compare whether there is a significant difference between the two impact factors on the spatial distribution of traditional villages, measured by F statistics:

$$F=\frac{{SSW}_{X_{a}}N_{X_{a}}(N_{X_{b}}-1)}{{SSW}_{X_{b}}N_{X_{b}}(N_{X_{a}}-1)}$$ (8)

$${\mathrm{SSW}}_{X_{a}}=\sum_{h=1}^{L_{a}}N_{h}\sigma_{h}^{2},{\mathrm{SSW}}_{X_{b}}=\sum_{h=1}^{L_{b}}N_{h}\sigma_{h}^{2}$$ (9)

Where, $N_{X_{a}}$ and $N_{X_{b}}$ are the sample sizes of the two independent variables *Xa* and *Xb* respectively; And represent the sum of the intralayer variances of the layers formed by *Xa* and *Xb* respectively; *La* and *Lb* are the number of layers of variables *Xa* and *Xb*, respectively. Where null hypothesis (H0): = ${SSW}_{X_{a}}={SSW}_{X_{b}}$. If H_0_ is rejected at the significance level of *α*, it indicates a significant difference in the impact of the two independent variables Xa and Xb on the spatial distribution of the attribute dependent variable Y.

## 3 Results and analysis

### 3.1 Spatial feature analysis

#### 3.1.1 Characteristics of spatial distribution types

To explain the spatial characteristics of traditional villages from a macro perspective, traditional villages can be regarded as point elements, which are usually distributed in three types: random, discrete and cohesive [10].

Using the spatial statistical analysis tool ArcGIS10.5 to perform traditional village nearest neighbor index analysis on the Puxian area unit. If *r*_*1*_ = 2008.07 and *r*_*E*_ = 2831.06 are obtained, then the ratio of the actual nearest distance mean to the theoretical nearest distance R = 0.71, resulting in R<1. Therefore, the spatial distribution of traditional villages in Puxian is a condensed distribution.

#### 3.1.2 Spatial distribution equilibrium

*(1) Distribution characteristics of counties*. The total number of Puxian traditional villages T is 137, and the total number of county (district) administrative units n is 5. Assuming that 137 traditional villages are evenly distributed in the five counties of Puxian region, the number of traditional villages that should be distributed in each county is 137 / 5 = 27.40, that is, the balanced geographical concentration index should be G_0_ = 27.40. By calculating the concentration function relationship, the geographical concentration index of Puxian traditional villages is G = 48.03. G_0_ is slightly less than the calculation result of 48.03, so the distribution of traditional villages at the county level is relatively concentrated, but the concentration degree is not high.

**Fig 2:**
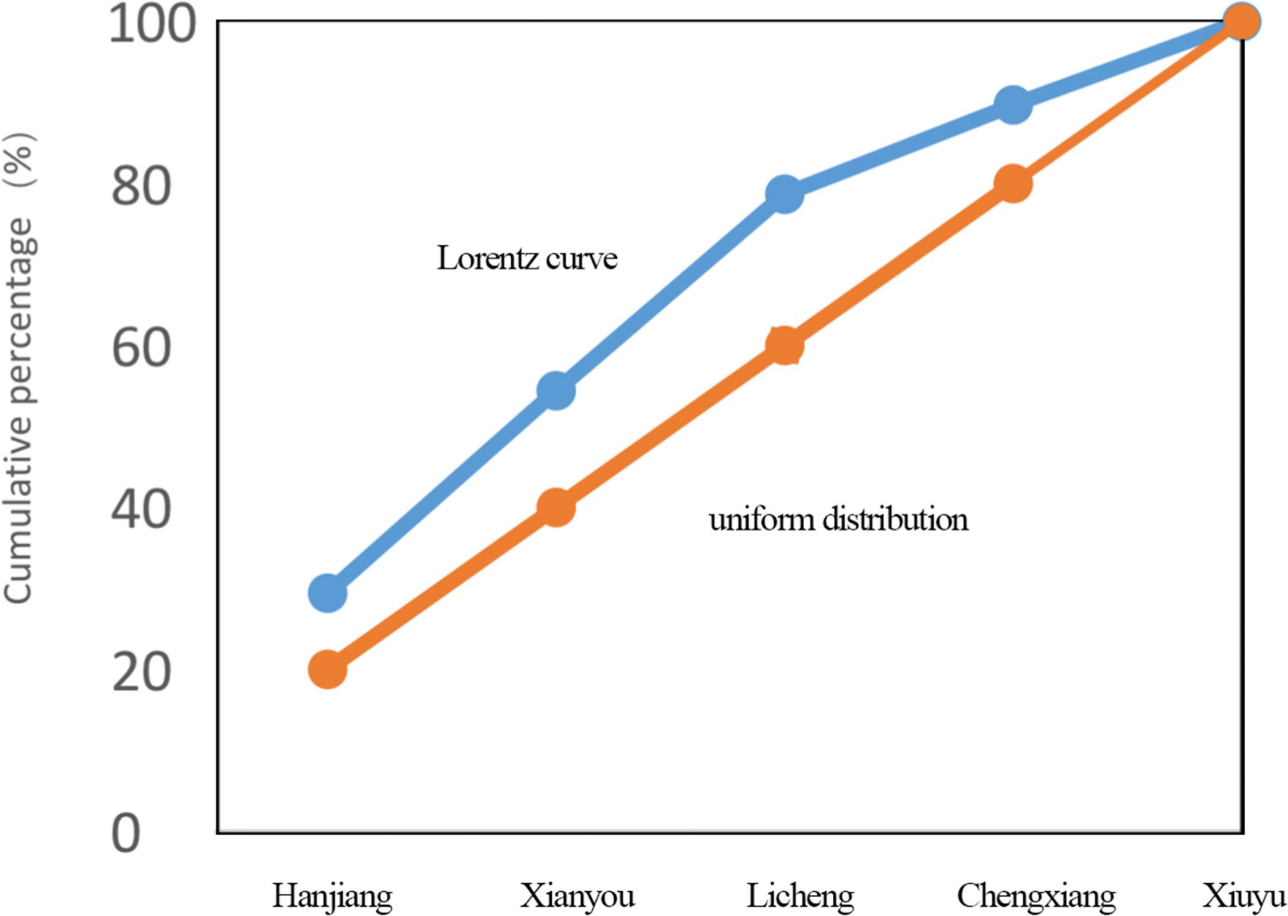
Lorentz curve of Puxian traditional village.

*(2) Spatial distribution equilibrium degree*. According to the structural relationship of spatial Gini coefficient, the spatial Gini coefficient of Puxian traditional villages is calculated, and the result is G = 0.32, indicating that Puxian traditional villages are concentrated and distributed in an uneven trend, mainly in Hanjiang District (29.41%). According to the imbalance index, the imbalance index of traditional villages is S = 0.26. From the meaning of the index, it can be seen that the distribution of traditional villages in Puxian county is uneven and concentrated. As shown in Fig 2, the Lorentz curve can further show the spatial distribution of traditional villages in each county. The traditional villages in Puxian are mainly distributed in Hanjiang District (29.41%), and less in Xiuyu district (10.29%).

**Fig 3:**
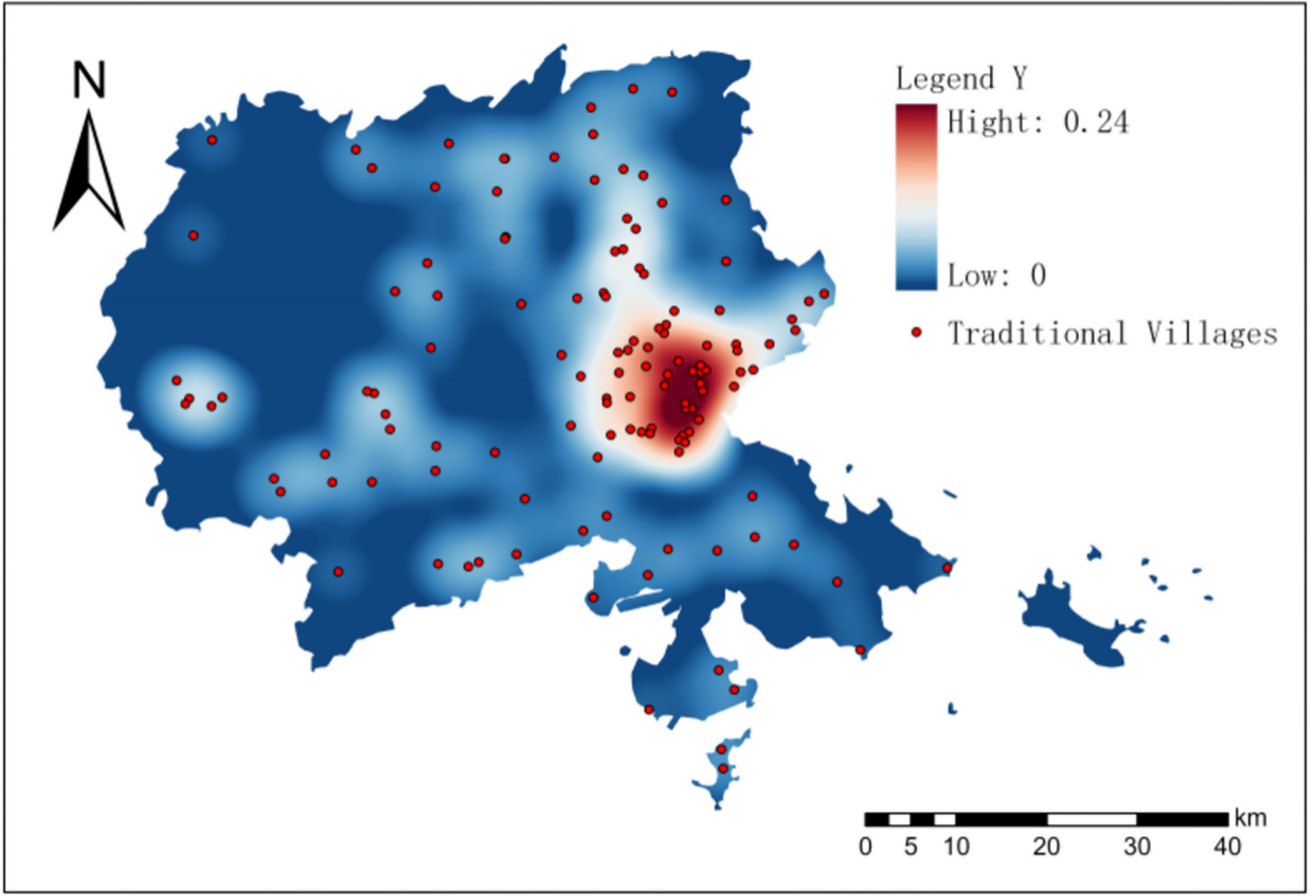
Spatial distribution and nuclear density distribution of Puxian traditional villages.

*(3) Spatial distribution pattern of traditional villages*. The nuclear density of 137 traditional villages in Puxian area was estimated by using the nuclear density tool in ArcGIS. According to Fig 3, a relatively high density concentration area was formed at the junction of Hanjiang District and Licheng District in the east of Puxian, and it was scattered in other areas. Overall, the spatial distribution pattern of "one cluster with more scattered points" with more coastal areas and less mountainous areas is presented.

### 3.2 Spatial heterogeneity analysis

#### 3.2.1 Selection of influencing factors

**Fig 4:**
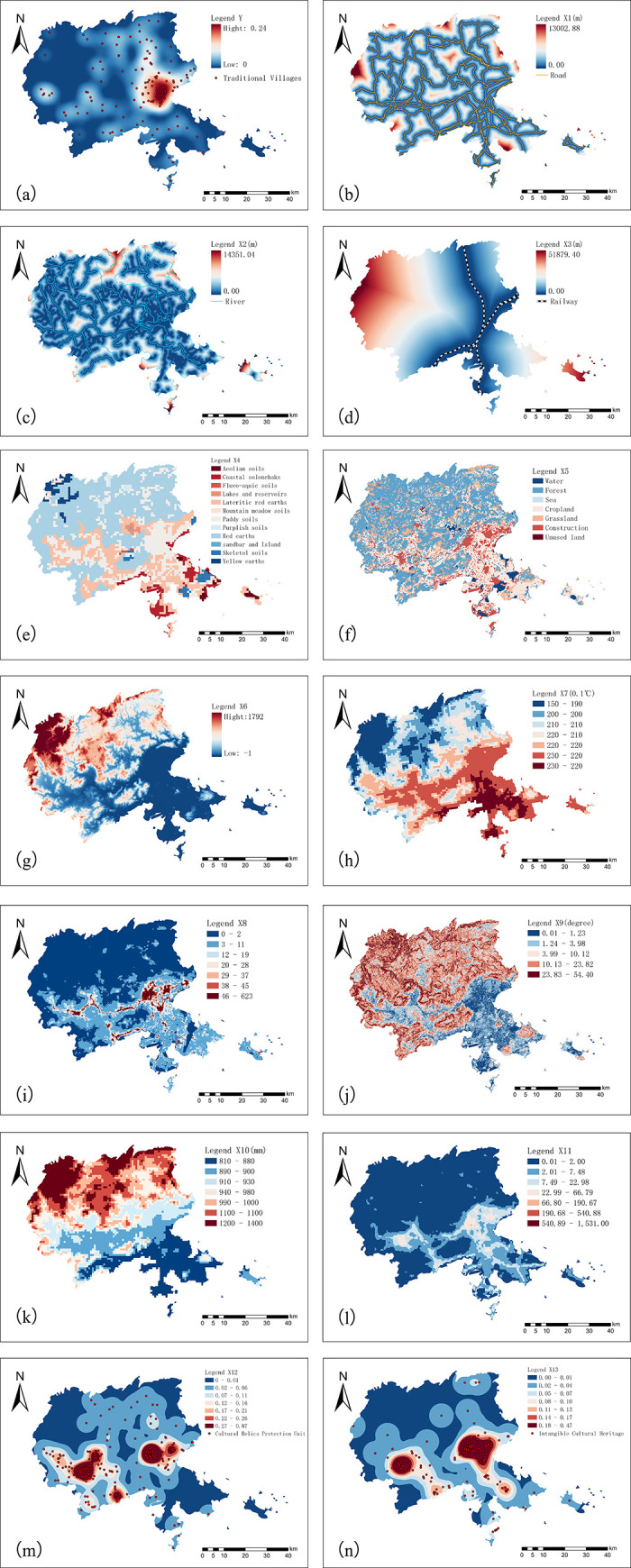
Puxian traditional village and its influencing factors coupling diagram.

The distribution of traditional villages is affected by many aspects, such as physical geography, social economy and so on. This study refers to the research results of Tong Yuquan [9], Ma Yong [51], Li Bohua [11], Li Yan [52], Li Jiangsu [10], Yu Jing [53], Guan Zhongmei [28], Liu Dajun [49], Dong Yanping [54] and so on in the selection of influencing factors. The above research mainly selected natural factors and economic and social factors, and the indicators include terrain, economy, population, transportation, climate, city, etc. The above study did not take into account the impact of natural ecological factors such as farmland and soil, cultural relics protection units and intangible culture on the spatial distribution of traditional villages. While drawing on previous results, this study added natural ecological factors, spatial factors and cultural factors. The final selected independent variables cover 13 factors, including soil type, land use, elevation, slope, temperature, precipitation, water system, railway, highway, population, economy, cultural protection unit and intangible cultural heritage (Fig 4).

#### 3.2.2 Determination of optimal spatial element and discretization method

This study intends to use geodetectors to explore the driving factors of the spatial distribution of traditional villages based on grid scale. Because the choice of different scales and discretization methods will affect the final q-value, this study uses four discretization methods, geometric, quantile, -tural and sd, based on R language, with 5–7 discontinuities. The best discretization method and discretization parameters of the best analysis scale are selected, and their values are as follows Table 2.

**Table 2:** Discretization parameter table.

Factor	Method	Number of discontinuities
X1	SD	7
X2	Quantile	7
X3	Quantile	7
X6	Geometric	7
X7	Quantile	7
X8	Geometric	7
X9	Geometric	6
X10	Quantile	7
X11	-Tural	5
X12	Quantile	7
X13	SD	7

**Fig 5:**
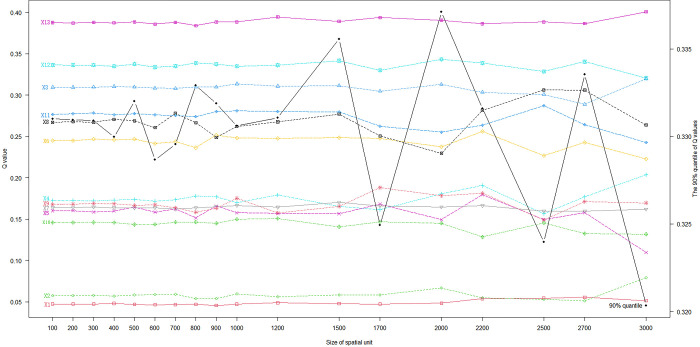
q-value change and discretization method selection basis under different scales.

This study explores the optimal analysis scale, uses the quantile discretization method, selects 7 discontinuities, and discretizes all continuous data. The results are as shown in the Fig 5: the fluctuation range of q-value under 90% quantile within 1000m is not large, but after 100m, its q-value fluctuates greatly with the scale, and reaches the maximum near 2000m. Therefore, 2000m grid is selected as the analysis scale in this study.

#### 3.2.3 Factor detector

**Fig 6:**
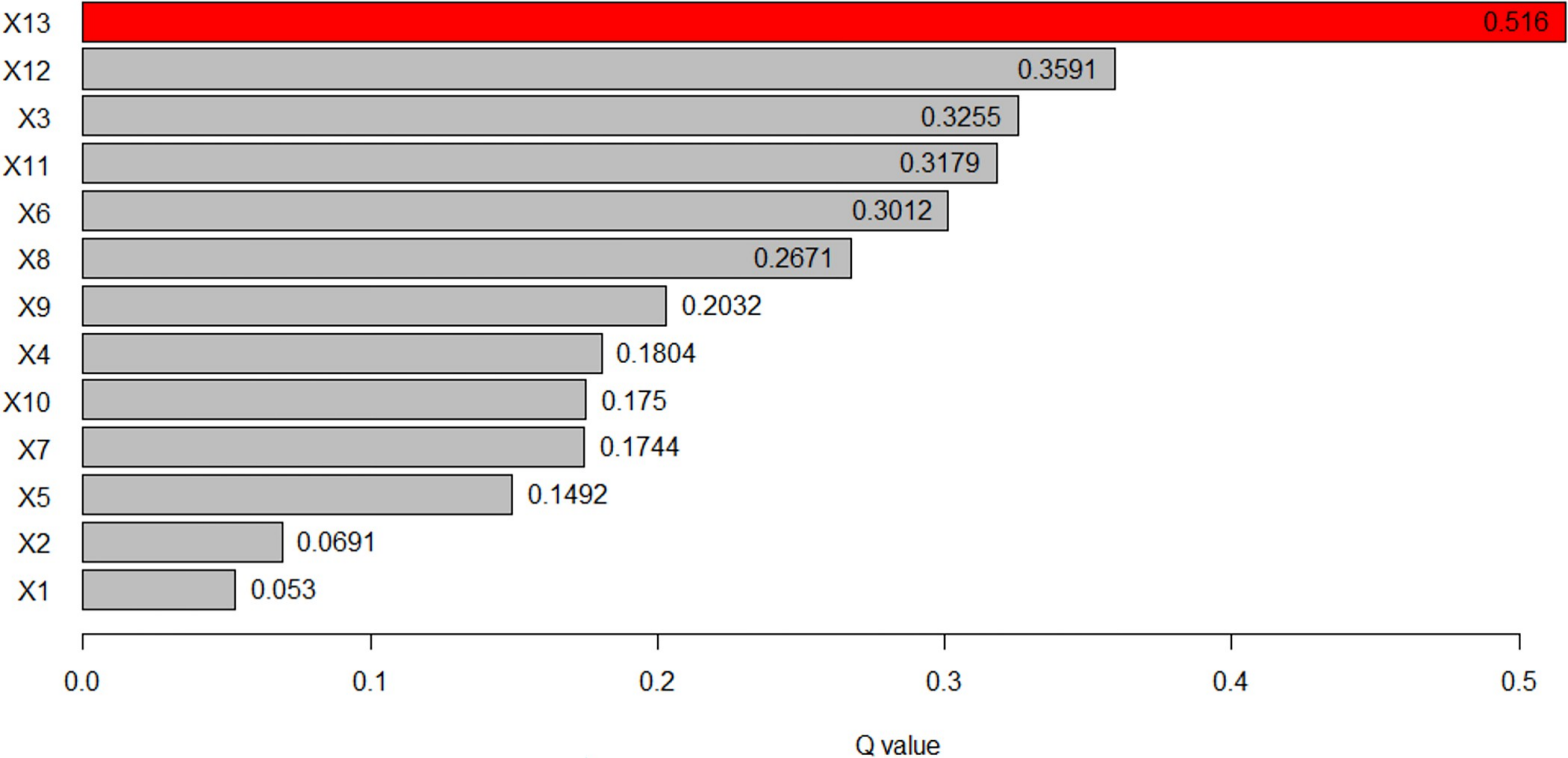
q-value ranking chart.

Using the GeoDetector software to analyze the influence intensity of factors, the concentration degree of traditional villages is taken as the independent variable Y to detect the degree of action of the driving factors affecting the concentration degree. Results as shown in Table 3, each indicator factor has a significant impact on the distribution of traditional villages in the study area, and all pass the 1% significance level test, the order was intangible cultural heritage (0.5160) > cultural protection unit (0.3591) > distance from railway (0.3255) > night light remote sensing (0.3179) > elevation (0.3012) > population density (0.2671) > slope (0.2032) > soil type (0.1804) > precipitation (0.1750) > temperature (0.1744) > land use (0.1492) > distance from river (0.0691)>distance from highway (0.0530) (Fig 6). That is, the influence of cultural factors such as intangible cultural heritage and protected cultural relic units on the spatial distribution of traditional villages in Puxian area is greater than that of natural, spatial and social factors.

**Table 3:** Statistical table of dominant driving forces and significance.

Independent Variables	Detection factor	Indicator factors	q-Value	Sig
X1	road	distance from highway	0.0530**	0.0000
X2	water system	distance from river	0.0691**	0.0000
X3	road	distance from railway	0.3255*	0.0000
X4	soil	soil type	0.1804*	0.0000
X5	land	land use	0.1492*	0.0000
X6	terrain	elevation	0.3012*	0.0000
X7	climate	air temperature	0.1744*	0.0000
X8	population	population density	0.2671*	0.0000
X9	terrain	slope	0.2032*	0.0000
X10	climate	precipitation	0.1750*	0.0000
X11	economic	night light remote sensing	0.3179*	0.0000
X12	protected cultural relic units	protected cultural relic units	0.3591*	0.0000
X13	intangible cultural heritage	intangible cultural heritage	0.5160*	0.0000

Note

* * *, ** *, * respectively indicate that the variables are significant at 1%, 5% and 10% levels.

#### 3.2.4 Interaction detection

**Fig 7:**
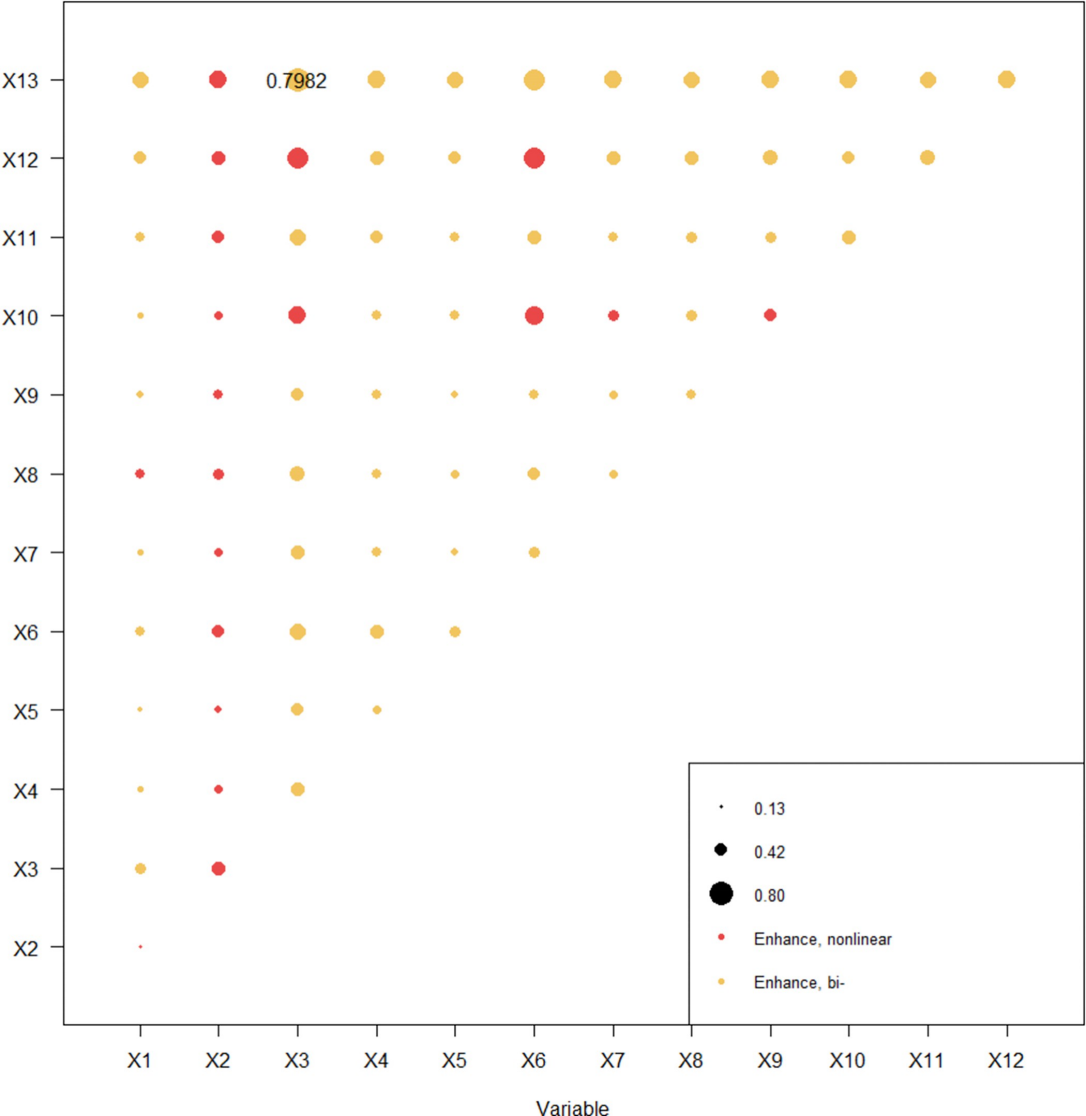
Interaction factor detection map.

**Fig 8:**
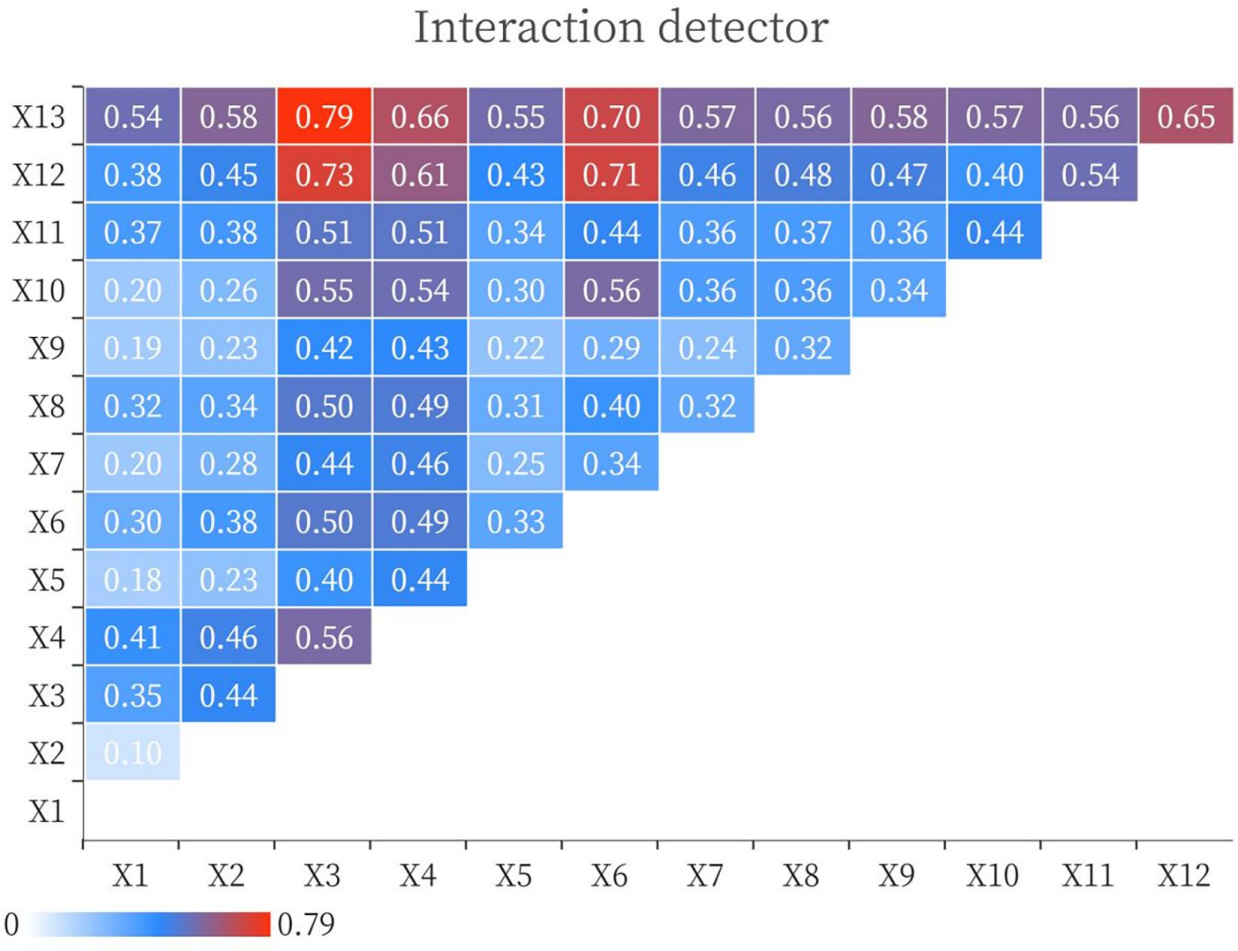
Interaction factor detection map.

The spatial distribution of traditional villages is often affected by a variety of factors, and a single factor cannot exist or affect the distribution or change of traditional villages [55]. Therefore, analyzing the interaction degree of various factors on the spatial distribution of traditional villages through the Interaction Detector helps to accurately determine the deep driving mechanism affecting the spatial distribution of traditional villages [56]. It can be seen from Figs 7 and 8 that the interaction of two factors can explain the spatial differentiation of 137 traditional villages more than that of single factor, most of which are double factor enhancement, accounting for 48/66, and a few are nonlinear enhancement, accounting for 18/66. There is no independent or weakening type. The interaction value between intangible cultural heritage and the distance from the railway is the highest, followed by the interaction value between protected cultural relic units and the distance from the railway.And the interaction value of intangible cultural heritage and protected cultural relic units with other factors is not less than 0.38, among which the interaction value of intangible cultural heritage ∩ distance from the railway is the strongest, with a q-value of 0.79. In general, the interaction of cultural factors and other factors has an important impact on the spatial differentiation of Puxian traditional villages, and natural, spatial and social conditions are also important factors affecting the distribution of traditional villages.

#### 3.2.5 Risk area detection

**Fig 9:**
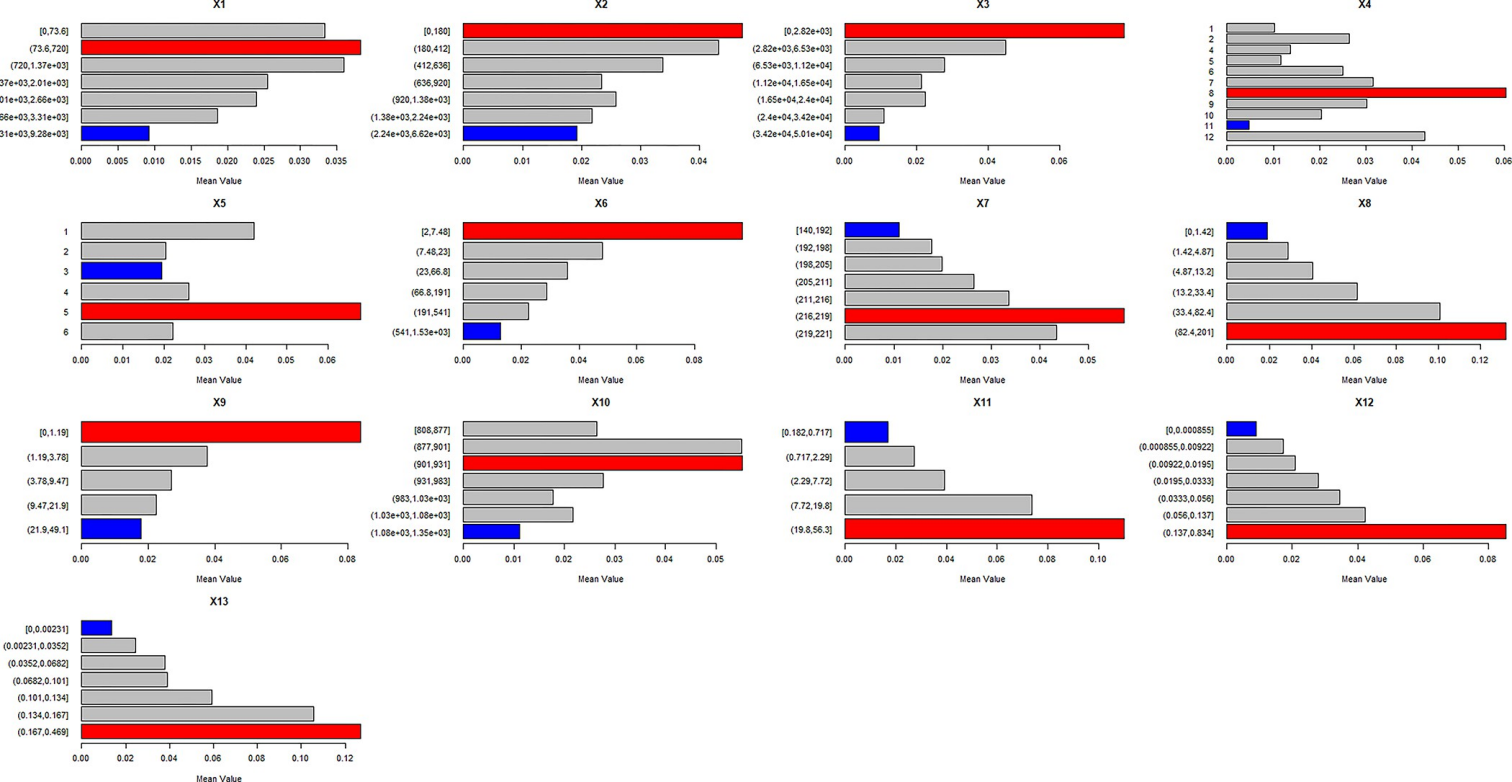
Detection results of risk area.

The risk area detector was used to detect whether there were significant differences in attribute values between each two sub areas of 13 factors (that is, each category of each independent variable) and whether the traditional villages were in the high value area and low value area of each factor sub area. It can be seen from Fig 9 that the significant differences in intangible cultural heritage sub regions are strong, and most sub regions show significant differences. The contribution rate of each source is different in each region after the division of continuous variables. Among them, the regions with high contribution rates are 73.6-720m away from the highway, 0-180m away from the river, 0-280m away from the railway, soil types 8 (paddy soil), land uses 5 (construction land), 2–7.48m in elevation, 21.6–21.9°C in temperature, 82.4–201 people / km2 in population density, 0–1.19°in slope, 901-931mm in annual precipitation, 19.8–56.3cd/m2 in night light remote sensing. The nuclear density of protected cultural relic units is 0.137–0.834bps, and the nuclear density of intangible cultural heritage is 0.167–0.469bps. In addition, the analysis shows that the results of risk area detection are consistent with the results of factor detection, that is, the factor detection has a strong explanatory power on the distribution of traditional villages, and there are significant differences between its sub regions.

#### 3.2.6 Ecological detection

**Fig 10:**
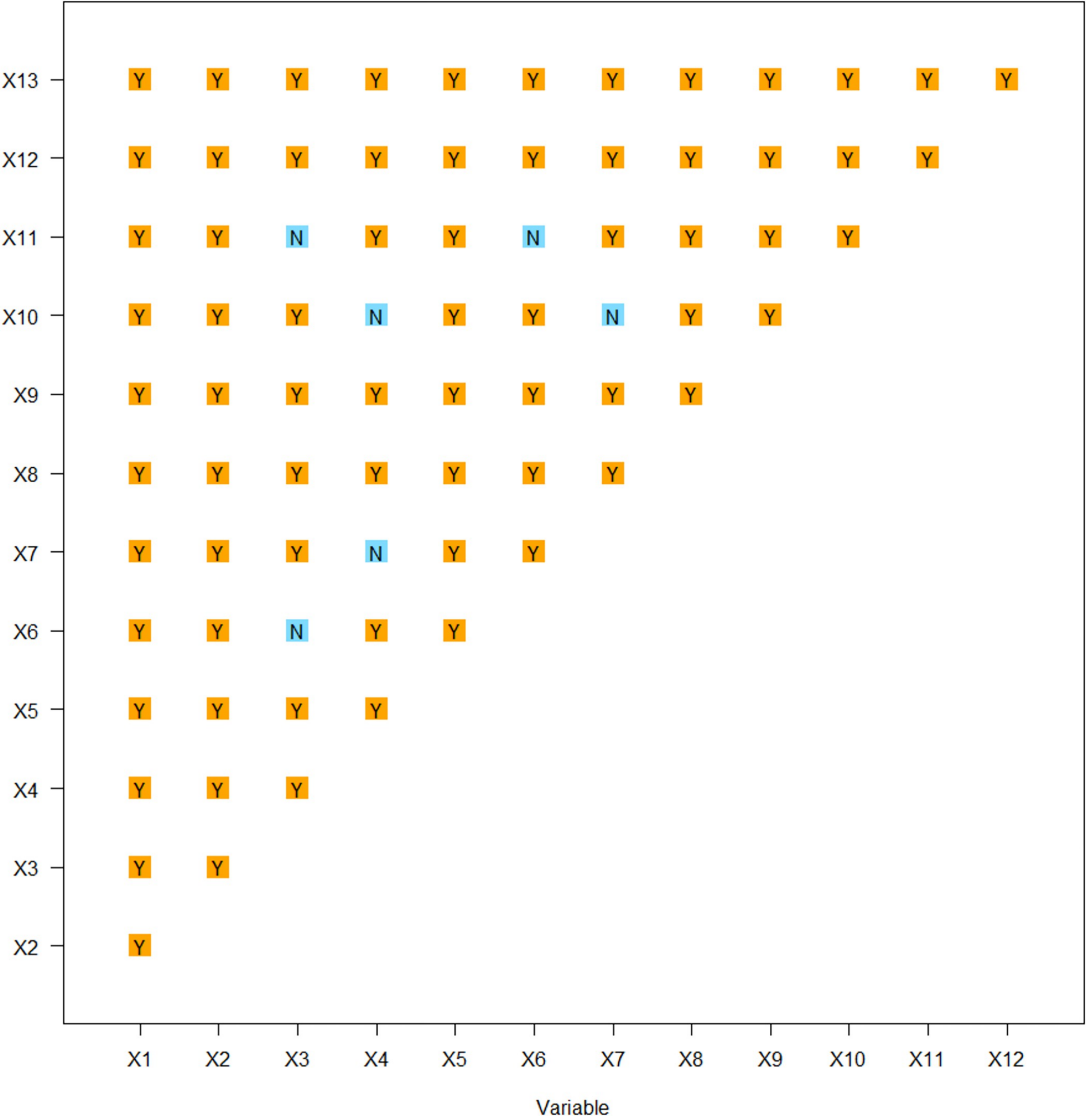
Ecological detection results.

Ecological detection focuses on comparing whether each two independent variables (impact factors) have significant differences in the impact on the spatial distribution of dependent variables (Puxian traditional villages) [56, 57]. If significant, it is recorded as Y, otherwise it is recorded as N. The ecological detection results of traditional villages in the study area show that there is no significant difference between the distance from the railway and elevation and night light remote sensing, soil type and temperature and precipitation, elevation and night light remote sensing, temperature and precipitation, while there are significant differences in the impact of other factors on traditional villages (Fig 10).

### 3.3 Analysis of influencing factors of spatial distribution

The influencing factors are discretized by quantile discretization method, and the analysis scale is selected. Then, with the help of GIS overlay analysis, geodetector and other methods, the specific influence law of each factor is further analyzed (Table 4).

**Table 4:** Influencing factors of Puxian traditional village.

Index factor	Number	Standard of classification	Classification result		
			Name	Quantity (units)	Proportion (%)
distance from highway	X1	Natural	0-1km	96	70.1
			1-2km	25	18.2
			2-3km	17	12.4
			3-4km	7	5.1
			4-5km	7	5.1
			> 5km	0	0
distance from river	X2	Natural	0-1km	96	70.1
			1-2km	24	17.5
			2-3km	8	5.8
			3-4km	4	2.9
			4-5km	3	2.2
			>5km	2	1.5
distance from railway	X3	Natural	0-1km	26	19.0
			1-2km	25	18.2
			2-3km	7	5.1
			3-4Km	6	4.4
			4-5km	6	4.4
			> 5km	65	47.4
soil type	X4	[0–0.05) bps is a low density concentration area	low density concentration area	red earth	54.1
		[0.05–0.15) bps is a medium density concentration area	medium density concentration area	red earth	34.3
		[0.15–0.24) bps is a high-density concentration area	high density concentration area	paddy soil	90
land use	X5	Natural	cultivated land	67	48.9
			woodland	14	10.2
			meadow	6	4.4
			land used for building	49	35.8
elevation	X6	-1-50M is the low altitude, 51-200m is medium altitude, 201-500m is higher than the average altitude, 501-1000m is high altitude, 1001-1792m is extremely high altitude	low altitude	106	77.4
			medium altitude	18	13.1
			above average altitude	13	9.5
air temperature	X7	<20°C is a low temperature area	low temperature zone	3	2.2
		20–21°C is a medium temperature area	moderate temperature zone	21	15.3
		>21°C is high temperature area	high temperature zone	113	82.5
population density	X8	<20 persons /km2 is a low population density area	low population density area	24	17.5
		30–200 people /km2 is an area with medium population density	medium population density area	47	34.3
		>200 people /km2 is a high population density area	high population density area	66	48.2
slope	X9	0–5°	Grade I	105	76.6
		5–10°	Grade II	25	18.2
		10–15°	Grade III	6	4.4
		15–25°	Grade IV	1	0.8
		25–68°	Grade V	0	0
precipitation	X10	<900mm is a low precipitation area	low precipitation area	55	40.1
		900-1000Mm is the moderate precipitation area	moderate precipitation area	47	34.3
		>1000mm is high precipitation area	high precipitation area	35	25.5
night light remote sensing	X11	7.99–17.45cd/m2 is an economically underdeveloped area	economically underdeveloped areas	85	62.0
		0.15–7.98cd/m2 is an economically developed area	economically developed regions	23	16.8
		17.46–75.76Cd/m2 is an economically developed region	economically developed regions	29	21.2
cultural relics protection unit	X12	Natural	>3	101	73.7
			≤ 3	36	26.2
intangible cultural heritage	X13	Natural	>3	92	67.2
			≤ 3	45	32.8

#### (1) Analysis of natural factors

Soil Type (X4): Soil has an important influence on the location and layout of traditional villages. The soil types in Puxian area are mainly paddy soil and red soil, among which paddy soil is one of the important cultivated soils in China, which is formed by human production activities. The traditional villages in Puxian are mainly distributed in high-density areas with paddy soil types, accounting for 90% of all kinds of soil types in high-density concentrated areas. It can be seen that villagers prefer land suitable for agricultural production as the site for village construction.

Land Use (X5): Land use has always influenced the development and changes of traditional villages. The land use type of traditional villages in Puxian area is mainly cultivated land, accounting for 48.9%, followed by construction land, accounting for 35.8%. According to the distribution of traditional villages, the high-density concentrated areas are mainly cultivated land, which shows that villagers prefer to choose areas that can provide water sources and irrigation facilities for farming and living.

Elevation (X6): As altitude and slope increase, villages are less likely to settle. Villagers are better suited to live in low altitude plains, while high altitude areas are preferred by settlers due to their better defensive capabilities. In different elevation ranges of Puxian, the number of traditional villages in the plain area (<200 m) accounts for 77.4%, that in the hilly area (200–500 m) accounts for 13.1%, and that in the mountainous area (>500 m) accounts for 9.5%. As the altitude increases, the number of traditional villages gradually decreases.

Slope (X9): A proper slope is conducive to drainage, but not conducive to construction, agricultural production, housing, and many other factors. In the Puxian area, villages with slopes between 0–10° account for 94.8% of the total, villages with slopes less than 5° account for most, and traditional villages are not distributed in areas with slopes > 25°.

Climate (X7, X10): Climate is an important factor in the location of villages, and the influence of temperature is stronger than that of precipitation. The northwest of Puxian area is mountainous, and the temperature is low. Temperature is one of the necessary conditions for carrying out agricultural activities. The density of traditional villages increases as the temperature increases from the northwest to the southeast; flooding and waterlogging occur frequently in the Puxian area, the precipitation gradually decreases from the northwest to the southeast, and the density of traditional villages increases as the precipitation gradually decreases. This shows that the western region has low temperature, large precipitation and fewer agricultural villages, while the eastern region has suitable climate and moderate precipitation, which promote agricultural development and are more suitable for people to build villages and agricultural production and life.

**Fig 11:**
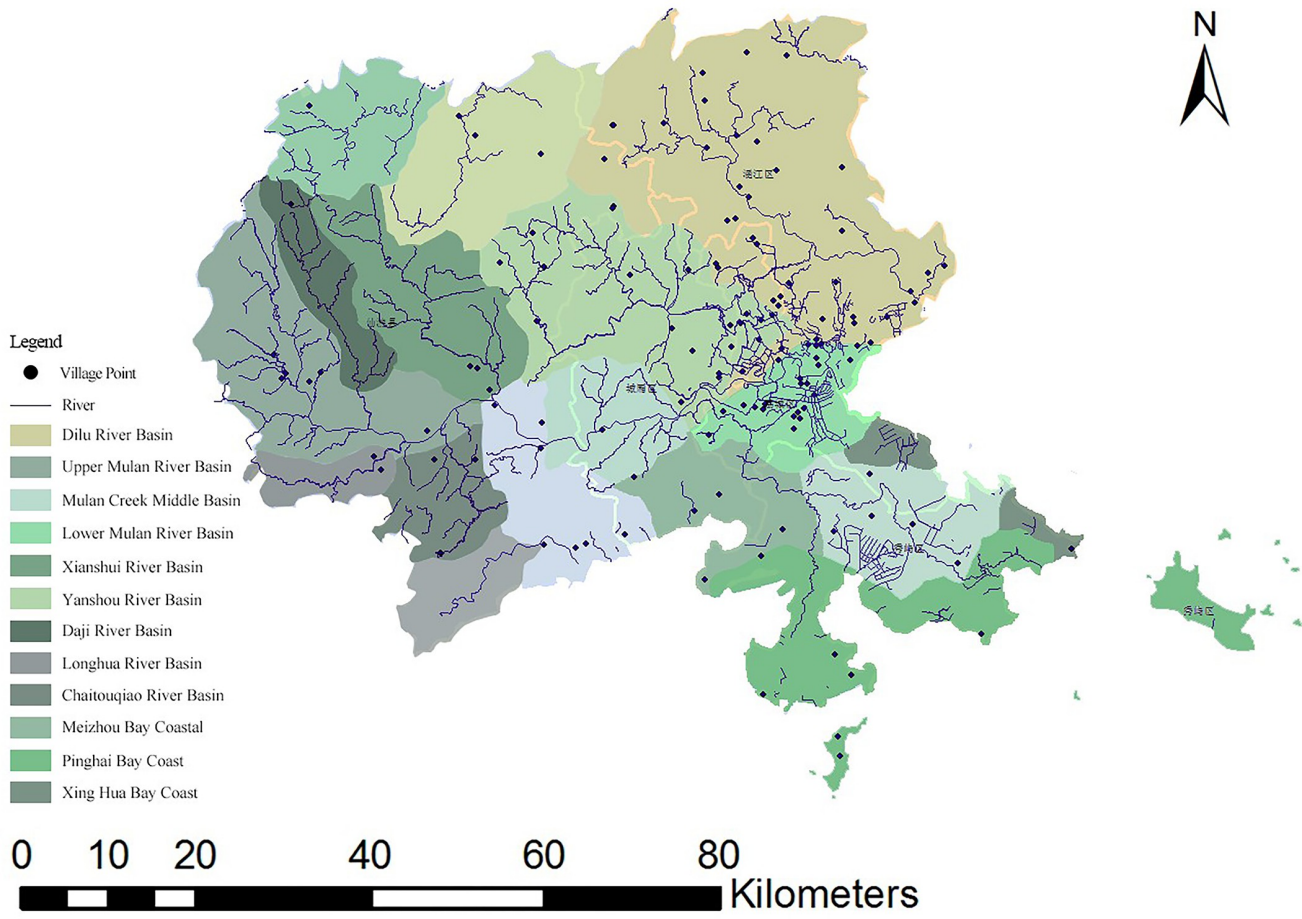
Distribution of traditional villages in each basin.

Water system (X2): The water system network is the carrier of the most rapid spread and development of civilization, providing important water sources and water transportation conditions for villages. The study area includes Mulan River, Daji River, Longhua River, Chaiqiaotou River, Xianshui River and Yanshou River. The water network in the eastern region is denser than that in the western region, which is a favorable guarantee for the development of traditional villages in the east. Compared with the east, Xianyou County in the west is prone to flood disasters due to its large annual precipitation and low river network density. Therefore, in order to survive safely, the distribution of traditional villages in the western region is smaller, and most of the villages are located at a certain distance from the mainstream. It can be seen from Table 4 that the area with the largest number of traditional villages is in the 1000m buffer zone, a total of 96, accounting for 70.1% of the total. The number of traditional villages in the 1000-2000m buffer zone is 24, accounting for 17.5%. As the distance of the buffer zone increases, the number of traditional villages shows an overall downward trend, and there is a significant clustering phenomenon in the lower reaches of the Mulan River, Yanshou River, and Dilu River basins (Fig 11).

#### (2) Spatial factor analysis

Roads (X7, X10): As the main transportation condition between rural settlements, roads provide a channel for material and information exchange for traditional villages. They can guarantee the long-term development of traditional villages and are the basic support for regional social and economic development. As shown in Table 2, traditional villages are most distributed within 1000 meters, with roads accounting for 70.1% of the total and railways accounting for 19.0% of the total. As the distance from the main roads increases, the number of traditional villages tends to decrease. From the perspective of transport modes, the coverage rate of roads to traditional villages within the same buffer distance is significantly higher than that of railways, and the spatial distribution of traditional villages is more dependent on road transport. This is related to the fact that under the background of the increasing demand for daily commuting convenience, road transportation has become the preferred mode of transportation for people with the advantages of high accessibility and high flexibility. Therefore, strengthening the construction of roads connecting traditional villages will help promote the layout and development of traditional villages.

#### (3) Analysis of social factors

Population (X8): The Puxian area is inhabited by 48 ethnic groups, including Han, Hui, Miao, Tujia, Yi, Buyi, She, Zhuang, Dong, Bai, and so on. The residents of She, Hui and other ethnic minorities are distributed in areas of high population density. Under the influence of multi-ethnic integration, they integrate Mazu culture, Central Plain culture, and Minyue culture, and develop traditional villages with unique regional style. With the gradual increase of population density, the distribution density of traditional villages also increases, indicating that the distribution of traditional villages is greatly influenced by population flow, which promotes the spread of culture, and there is a positive relationship between the two. This is because the good regional culture has laid a solid foundation for the formation, development and protection of traditional villages due to the strong regional characteristics of ethnic minorities, diverse customs and rich historical resources.

Economy (X11): The night light can clearly reflect the economic development level of Puxian area. The night light in the study area shows a decreasing trend from southeast to northwest. Puxian traditional villages are more distributed in areas with low economic development, while areas with low economic development are less affected by urbanization, which makes the traditional village culture more intact.

#### (4) Analysis of cultural factors

Protected Cultural Remnants (X12): Protected cultural relic units reflect the historical and cultural connotation of a region. The more concentrated areas have a more profound history, laying a cultural foundation for the rise and sustainable development of traditional villages. The protected cultural relics units in Puxian area are concentrated in the east and west sides of the central part. The main types of protected cultural relics units in this area are ancient buildings and historical sites, which are related to historical and economic factors and trade development factors. Compared with other influencing factors, the concentration of protected cultural relics units has the greatest correlation with the distribution of traditional villages, which shows a highly positive correlation. Villages and protected cultural relic units have a certain complementary effect, and the research and construction of traditional village culture and characteristic landscapes can contribute to the development of famous traditional cultural heritage villages.

Intangible Cultural Heritage (X13): The intangible cultural heritage in Puxian area is concentrated in the east and west sides of the central region. Licheng District, Xianyou County and other areas are highly concentrated. Many villages rely on intangible cultural heritage to promote economic development.

## 4 Discussion and conclusion

### 4.1 Discussion

Puxian traditional village carries the architectural concept of harmony between man and nature and the essence of local culture formed in the traditional ritual society [58, 59], whose layout emphasizes seeking advantages and avoiding disadvantages, making rational use of the natural environment, and respecting the landscape texture and natural laws [60], reflecting the traditional wisdom and experience of the coordinated coexistence of human habitat and nature in the long historical process. In previous studies, Xiang Xu et al. (2022) [61] found that terrain is no longer the dominant factor in the development of villages, and natural, economic, cultural and other factors jointly affect the development of villages. Yin Wei et al. (2022) [62] studied the spatial distribution characteristics of Chinese traditional villages in the Shu cultural corridor (Chengdu-Chongqing region), and found that natural factors affected the origin, human factors affected the development, and various factors jointly restricted the development. Compared with the existing results, this study found that natural factors influenced the independent formation of the spatial pattern of Puxian village, and determined the overall differentiation pattern of traditional villages.Spatial factors affect the evolution and development pattern of traditional villages, and play a fundamental role in the spatial distribution pattern of traditional villages. Social factors, as the external driving force affecting the spatial differentiation of Puxian traditional villages, are the dominant factors determining the distribution of traditional villages. Cultural factors have laid a cultural foundation for the rise and sustainable development of traditional villages. The most important difference is that the study area is geographically and scenically diverse, leading to changes in village locations, as well as cultural and economic differences, which promote or hinder the selection and development of village locations.

The Puxian region is characterized by mountains, rolling hills, intertwined valleys and gullies, vast fertile plains, and wide and long sea areas, forming a complex and diverse grid-like terrain. The traditional villages in the region are distributed in an uneven spatial pattern of "There are many villages in coastal plain areas, but few in inland mountainous areas" and "one clump with multiple scattered points".The distribution of traditional villages in Puxian is influenced by multiple factors, by analyzing the impact intensity of the factors, we found that intangible cultural heritage (0.5160) > protected cultural relic units (0.3591) > distance from railway (0.3255) > night light remote sensing (0.3179) > elevation (0.3012) > population density (0.2671) > slope (0.2032) > soil type (0.1804) > precipitation (0.1750) > temperature (0.1744) > land use (0.1492) > distance from river (0.0691) > distance from highway(0.0530). The interaction value between intangible cultural heritage and distance from the railway is the highest, followed by the interaction value between protected cultural relic units and distance from the railway. The interaction value between intangible cultural heritage and protected cultural relic units and other factors is not less than 0.38, among which the interaction value of intangible cultural heritage ∩ distance from the railway is the strongest, with a q-value of 0.79. The main reason is that the central part of Puxian is composed of low mountains and hills, with abundant water sources, intertwined plains and valleys, and rich soil quality, suitable for human habitation, production and reproduction, and cultural heritage. The southeast coast is surrounded by peninsulas and hilly terraces, surrounded by harbors, hills and terraces. Hanjiang District and Licheng District, where the villages are concentrated, are located in the estuary of the main stream of the Mulan River, as well as in the alluvial plain and marine plain. Hanjiang District and Licheng District have been sailing along the Silk Road and gathering merchants since ancient times. Licheng District was once part of Putian County, Xinghua Prefecture, and is one of the central urban and economic centers of Putian City. Hanjiang is a thousand-year-old city in Putian and one of the four important cities in Fujian. It is known as "Scenery Little Wu Yue, Wealth Jia Zhangquan", so it has gathered a large number of traditional villages with historical and cultural connotation. With the continuous improvement of the level of urbanization and modernization, the protection of the historical and cultural heritage of traditional villages has been continuously promoted, and the local economic conditions and the increasing ideology of residents have created conditions for the preservation of traditional cultural customs and production and lifestyle. While the western and northern regions are dominated by mountains, with low mountains, valleys and basins intermingled.The traffic development is relatively slow, and the social and economic development is self-sufficient, which is not easy to be affected by the outside world, and has certain advantages in protecting traditional villages; although the west and north are protected by mountains and forests to avoid outside interference, they are not suitable for production and life.

The planning of traditional villages should consider the spatial heterogeneity of different factors, identify the dominant factors affecting the development of each village through geodetectors, and formulate protection and development plans for different traditional villages, so as to improve the scientificity and adaptability of planning. For example, the traditional villages in Puxian area are strongly influenced by cultural factors. Intangible cultural heritage and protected cultural relics units have laid a cultural foundation for the rise and sustainable development of traditional villages. They reflect the diversity of Chinese culture in terms of architectural style, rural form and customs. In the future, we should avoid over-exploitation and safeguard the original cultural environment. In addition, conservation areas can be divided according to the degree of accumulation of Puxian traditional villages.

### 4.2 Conclusion

Taking 137 traditional villages in Puxian area as the research object, this paper explores their spatial distribution characteristics and driving factors, and draws the following conclusions.

Puxian traditional villages show an unbalanced spatial distribution pattern of "There are many villages in coastal plain areas, but few in inland mountainous areas" and "one clump with multiple scattered points". The junction of Licheng District and Hanjiang District is the area where villages are densely distributed.

Traditional villages in Puxian are mainly influenced by various factors such as nature, space, society, and culture. They are more densely distributed in areas with strong cultural heritage, fertile land, flat terrain, suitable climate, proximity to water systems, developed transportation, underdeveloped economy, and dense population.

The regional cultural characteristics of Puxian are distinct, and the different spatial distribution influencing factors of traditional villages show corresponding correlations, reflecting the degree and effect of each driving factor on the spatial distribution of villages.The factor detection results of 137 traditional villages and 13 influence factors at the county scale show that, intangible cultural heritage (0.5160) > cultural relics protection unit (0.3591) > distance from railway (0.3255) > night light remote sensing (0.3179) > elevation (0.3012) > population density (0.2671) > slope (0.2032) > soil type (0.1804) > precipitation (0.1750) > temperature (0.1744) > land use (0.1492) > distance from river (0.0691) > distance from highway (0.0530). That is, the influence of cultural factors such as intangible cultural heritage and protected cultural relic units on the spatial distribution of traditional villages in Puxian area is greater than that of natural, spatial and social factors. Interaction detection showed that the explanatory power of interaction was enhanced. The interaction between intangible cultural heritage and distance from railways is the dominant factor in the spatial differentiation of traditional villages, followed by the interaction between cultural relics protection unit and distance from railways.Risk detection found that the differences of intangible cultural heritage sub regions were the most significant, and the factors with greater explanatory power for traditional villages also had significant differences in the number of villages in the sub regions. Ecological exploration found that there were significant differences in the impact of other factors on traditional villages, except that there were no significant differences between the distance from the railway and elevation and night light remote sensing, soil types and temperature and precipitation, elevation and night light remote sensing, temperature and precipitation.

## Supporting information

S1 Data (ZIP)

S2 Data (ZIP)

S3 Data (ZIP)
