# Supplementary material for: A study on the spatial distribution characteristics and driving factors of traditional villages in the southeast coast of China: A case study of Puxian, Fujian
Source: PLoS One. 2024 Jun 7;19(6):e0303746. doi: 10.1371/journal.pone.0303746 (PMC11161066; doi:10.1371/journal.pone.0303746)
Supplement: S3 Data — (ZIP) [file pone.0303746.s003.zip › result data/result/data sources.docx]

data sources

| data | data sources | Website Address | resolution ratio |
| --- | --- | --- | --- |
| Land use data | Resources and Environment Science Data Center of Chinese Academy of Sciences | [www.resdc.cn](http://www.resdc.cn/) | 30m |
| Agricultural production potential | Resources and Environment Science Data Center of Chinese Academy of Sciences | [www.resdc.cn](http://www.resdc.cn/) | 1km |
| Soil type/erosion | Resources and Environment Science Data Center of Chinese Academy of Sciences | [www.resdc.cn](http://www.resdc.cn/) | 1km |
| GDP | Resources and Environment Science Data Center of Chinese Academy of Sciences | [www.resdc.cn](http://www.resdc.cn/) | 1km |
| precipitation | National Qinghai Tibet Plateau Scientific Data Center | [data.tpdc.ac.cn](http://data.tpdc.ac.cn/) | 1km |
| air temperature | National Qinghai Tibet Plateau Scientific Data Center | [data.tpdc.ac.cn](http://data.tpdc.ac.cn/) | 1km |
| Railways, highways | 《1: 1 million public version of basic geographic information data》  《1: 1 million public version of basic geographic information data》  《1: 1 million public version of basic geographic information data》 | [www.webmap.cn](http://www.webmap.cn/) | - |
| Nature Reserve |  | [www.webmap.cn](http://www.webmap.cn/) | - |
| Settlement |  | [www.webmap.cn](http://www.webmap.cn/) | - |
| population | World pop | [www.worldpop.org/](http://www.worldpop.org/) | 100m |
| NPP | MODISMOD17A | [https://lpdaac.usgs.gov](https://lpdaac.usgs.gov/) | 500m |
| DEM | ALOS | [https://search.asf.alaska.edu](https://search.asf.alaska.edu/) | 12.5m |
