# Supplementary figures and images for: A study on the spatial distribution characteristics and driving factors of traditional villages in the southeast coast of China: A case study of Puxian, Fujian
Source: PLoS One. 2024 Jun 7;19(6):e0303746. doi: 10.1371/journal.pone.0303746 (PMC11161066; doi:10.1371/journal.pone.0303746)

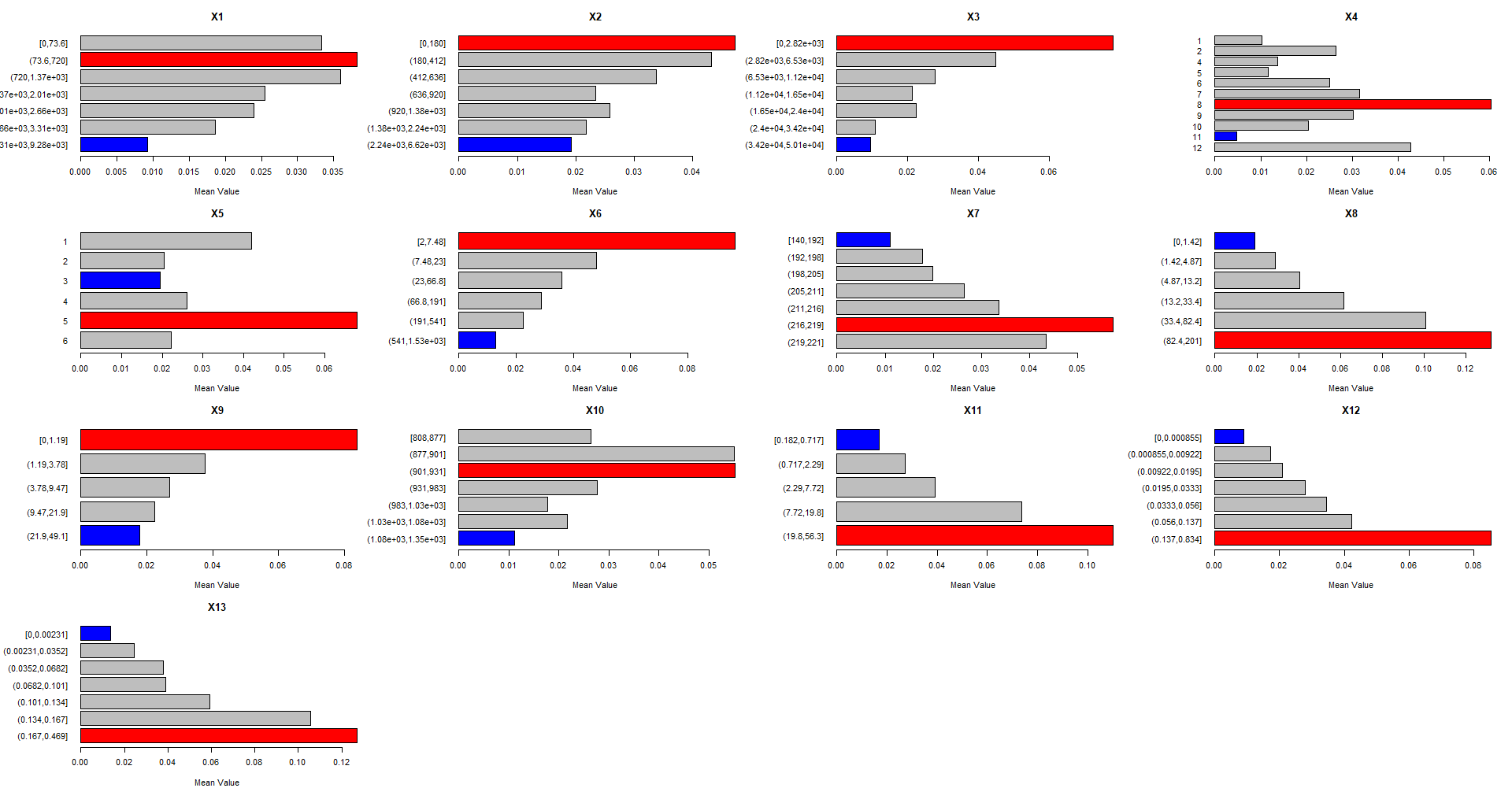

Supplement: S3 Data — (ZIP) [file pone.0303746.s003.zip › result data/result/Detection results of risk area.png]

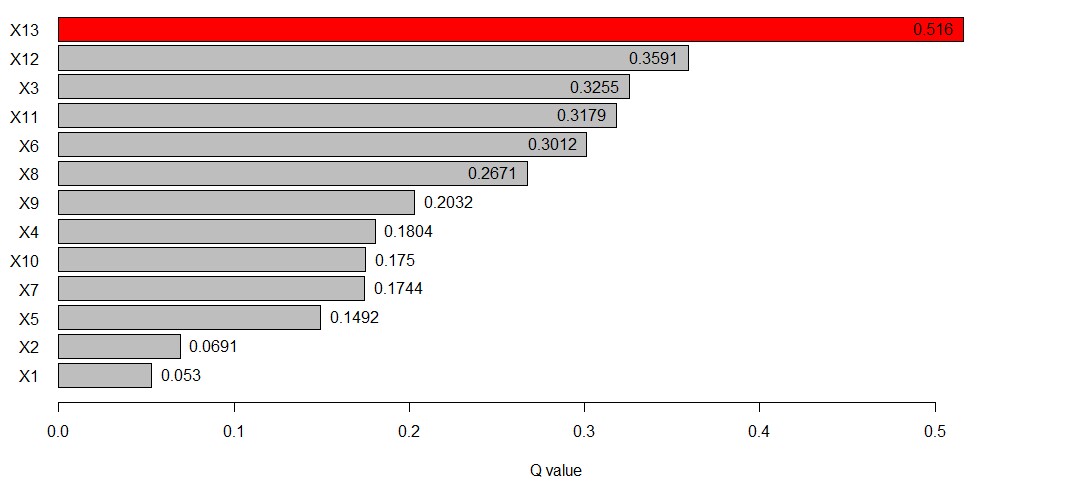

Supplement: S3 Data — (ZIP) [file pone.0303746.s003.zip › result data/result/Single factor detection.png]

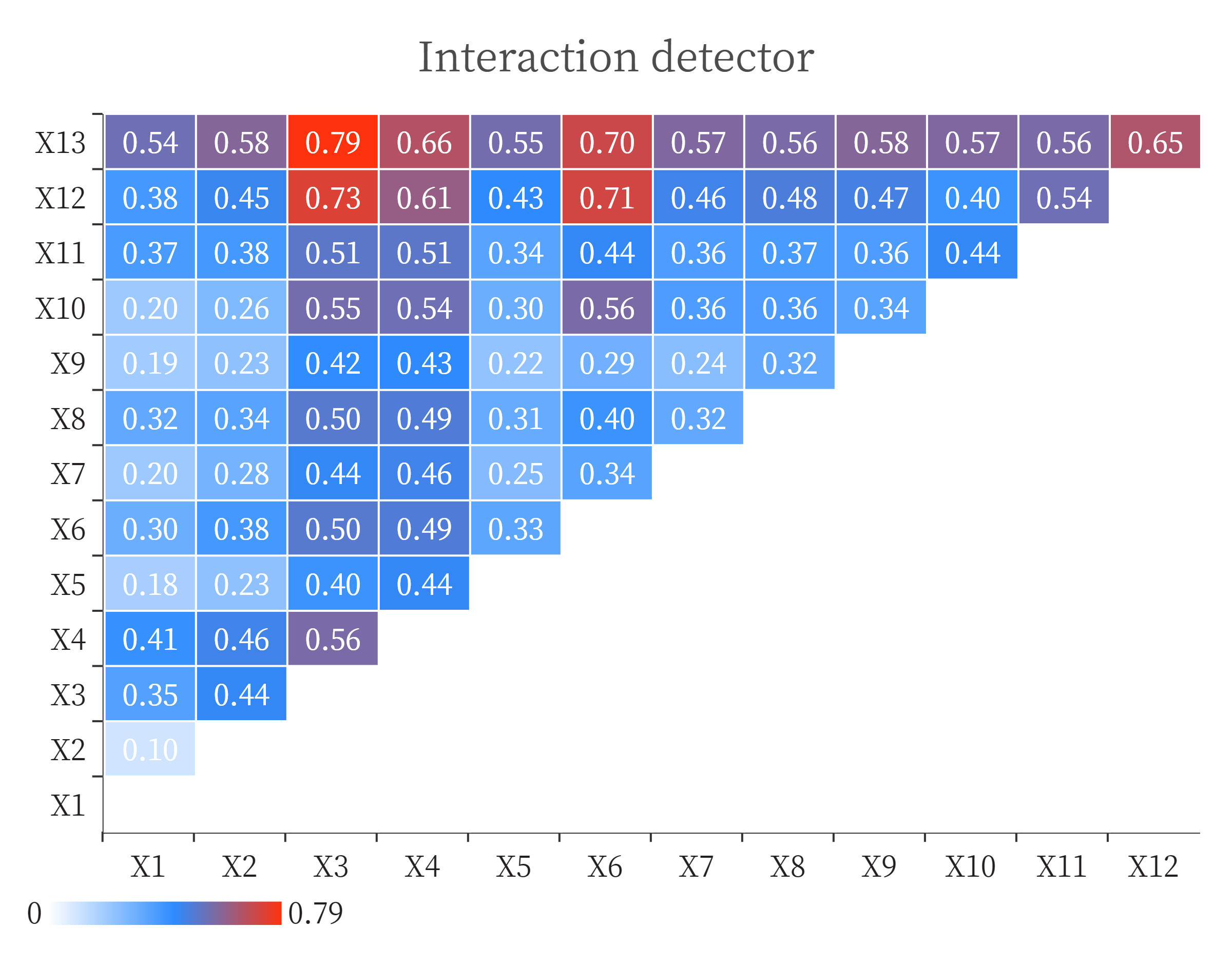

Supplement: S3 Data — (ZIP) [file pone.0303746.s003.zip › result data/result/interaction.jpg]

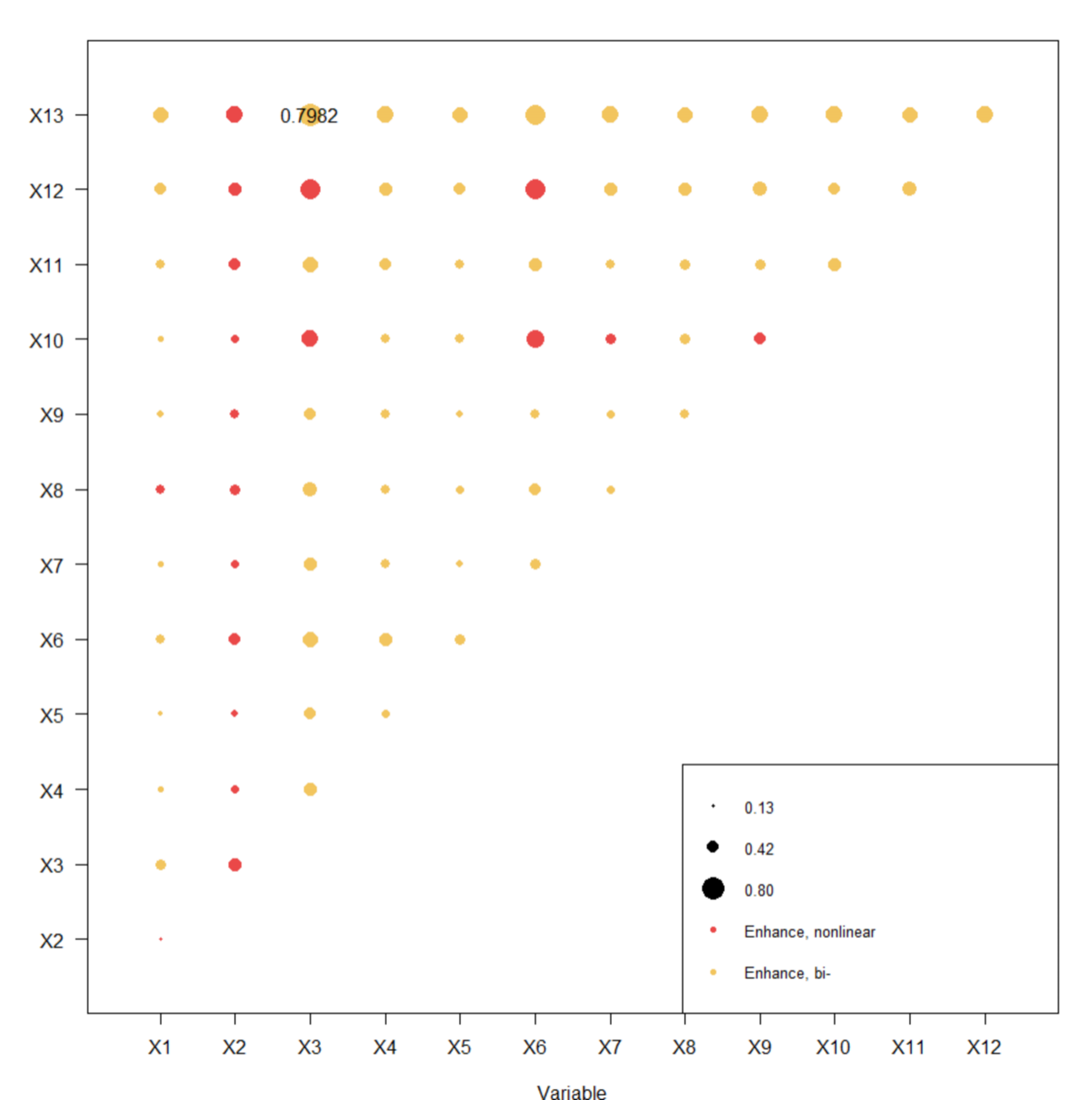

Supplement: S3 Data — (ZIP) [file pone.0303746.s003.zip › result data/result/Interaction map.png]

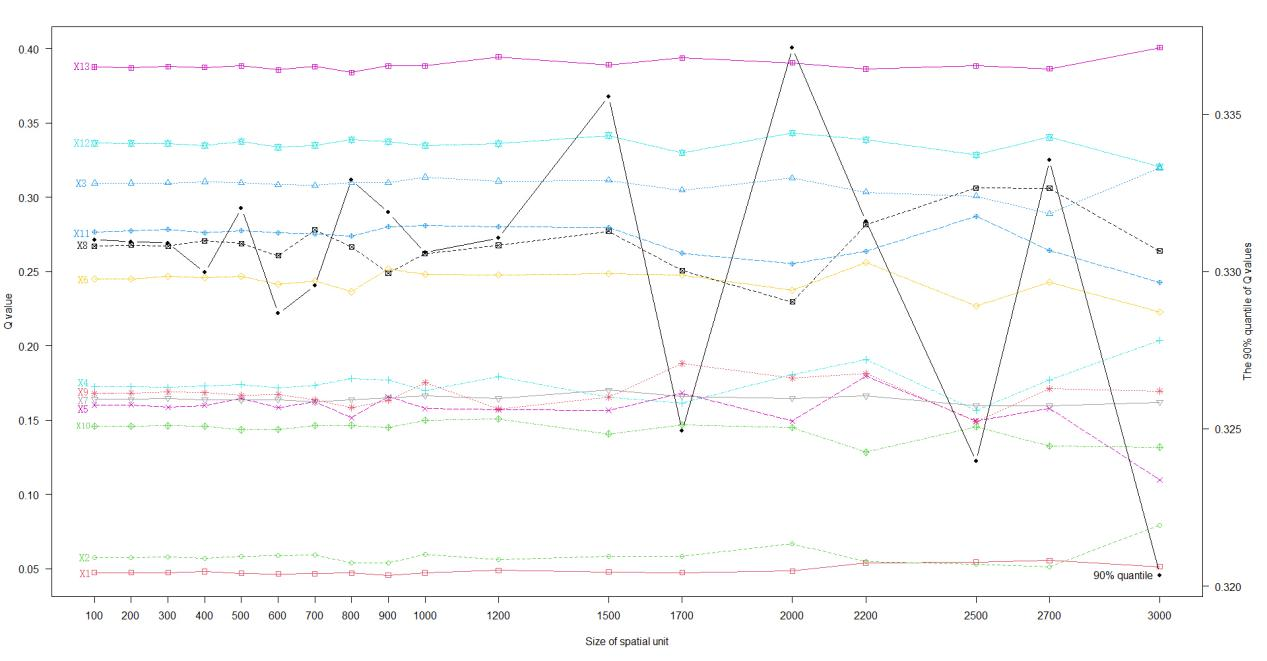

Supplement: S3 Data — (ZIP) [file pone.0303746.s003.zip › result data/result/quantitative Discretization.png]

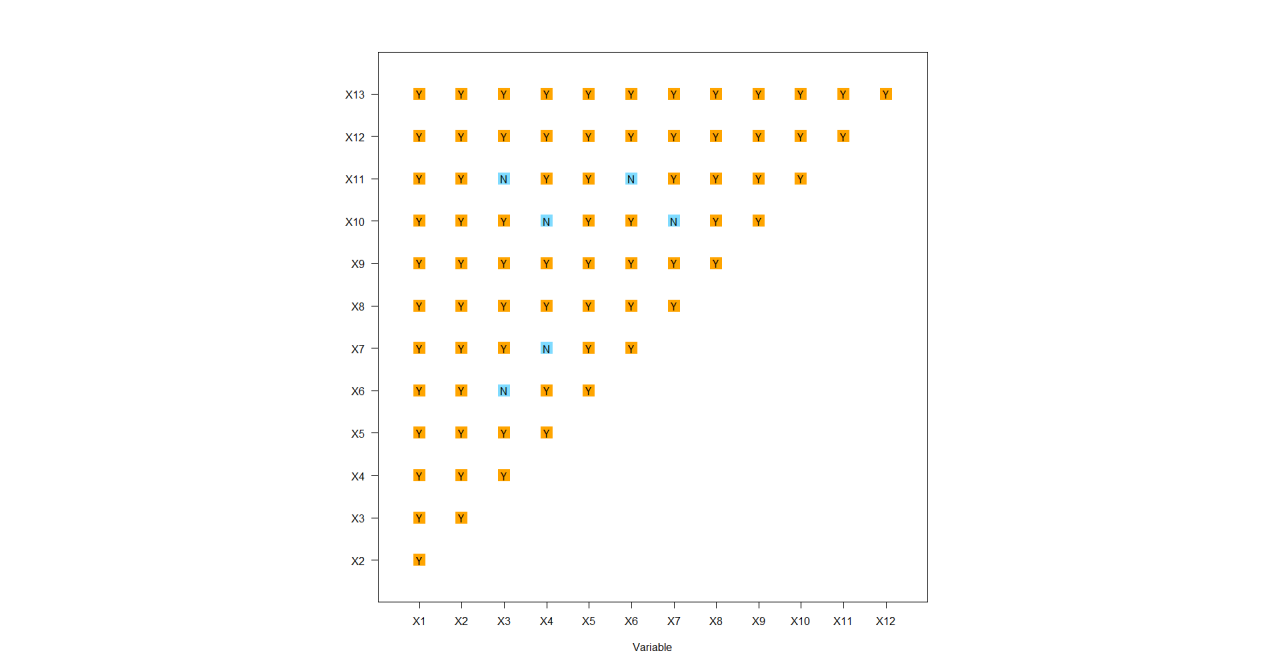

Supplement: S3 Data — (ZIP) [file pone.0303746.s003.zip › result data/result/Ecological detection results.png]
